# Mudança de Conduta em Pacientes de Fila de Cirurgia de Revascularização Miocárdica durante Pandemia de COVID-19: Seguimento de um Ano

**DOI:** 10.36660/abc.20220582

**Published:** 2023-04-12

**Authors:** Franc Jorge Sampaio Santos Pereira, Marília Prudente Menezes, Gabriela Carolina Santamaria Naranjo, José Henrique Herrmann Delamain, José Ribamar Costa, Mario Issa, Vivian Lerner Amato, Fausto Feres, Pedro Silvio Farsky

**Affiliations:** 1 Instituto Dante Pazzanese de Cardiologia São Paulo SP Brasil Instituto Dante Pazzanese de Cardiologia, São Paulo, SP – Brasil

**Keywords:** Doença da Artéria Coronariana/cirurgia, Revascularização Miocárdica, Intervenção Coronária Percutânea, Infarto do Miocárdio/complicações, COVID-19, Pandemia, Mortalidade, Listas de Espera, Tecnologia Aplicada a Listas de Espera

## Introdução

A pandemia de COVID-19 levou a uma importante ocupação de leitos de terapia intensiva, causando a suspensão dos casos eletivos de cirurgia de revascularização do miocárdio (CRM).

Ao mesmo tempo, a evolução de técnicas de tratamento percutâneo da doença arterial coronária (DAC) tem ampliado as possibilidades para essa modalidade, assim como estudos publicados não demonstraram redução de desfechos em pacientes estáveis com carga isquêmica moderada/alta mantidos em tratamento clínico otimizado (TCO).

Neste contexto, todos os pacientes incluídos em fila de CRM de um hospital terciário de cardiologia tiveram suas indicações revisadas por um *Heart Team* (HT) com o intuito de avaliar uma possível mudança de estratégia para intervenção coronariana percutânea (ICP) ou manutenção em TCO. Apresentamos as características clínicas e angiográficas, os motivos para mudança da estratégia terapêutica, assim como a evolução clínica após um ano de seguimento.

## Métodos

Todos os pacientes incluídos em uma fila de CRM entre junho de 2020 a abril de 2021 foram revistos por HT com especialistas sêniores em clínica, hemodinâmica e cirurgia, com o intuito de avaliar mudança de estratégia de tratamento. Pacientes com estudo angiográfico realizado há mais de um ano tiveram seu estudo repetido, assim como os de qualidade de imagem inadequada, ou na mudança significativa da sintomatologia. Pacientes pouco sintomáticos ou com indicação de revascularização miocárdica duvidosa foram submetidos a exames para quantificação de área isquêmica.

Os pacientes que tiveram a indicação cirúrgica modificada para outra modalidade de tratamento foram incluídos nesse estudo. Eles foram analisados quanto às comorbidades, anatomia coronariana e risco cirúrgico, assim como os motivos da mudança do manejo e evolução clínica no primeiro ano. Os pacientes foram acompanhados por uma equipe dedicada ao estudo e mantidos em tratamento médico otimizado.

Em relação aos desfechos clínicos, avaliou-se a incidência de mortalidade por todas as causas, mortalidade cardiovascular, infarto agudo do miocárdio (IAM), internação por causa cardiovascular, sangramento relevante e acidente vascular cerebral.

## Resultados

Dos 357 pacientes incluídos em fila de CRM entre junho de 2020 a abril de 2021, 43 (12,0%) foram excluídos pelo HT, sendo 21 submetidos a ICP e 22 mantidos em TCO. Os pacientes encaminhados a ICP tiveram maior prevalência de diabetes (57,1% x 36,3%) e IAM prévio (80,9% x 59%). Os mantidos em TCO tiveram maior prevalência de AVC prévio (13,6% x 0%) e maior risco cirúrgico conforme o escore STS (1,12% X 0,68%). Cinco pacientes (11,6%) apresentavam sintomas limitantes: três em NYHA III foram mantidos em TCO, um pelo elevado risco cirúrgico e outros dois por ter sido identificada doença pulmonar obstrutiva crônica como causa da dispneia; dois pacientes com angina CCS 3 foram encaminhados para ICP.

As características angiográficas são apresentadas na Tabela 1 .

**Tabela 1:** – Características angiográficas dos pacientes mantidos em tratamento clínico otimizado e submetidos a ICP

Artéria(s) acometida(s)	Clínico (n = 22)	ICP (n = 21)
Desc. Anterior	3 (13,6%)	3 (14,2%)
Biarterial	10 (45,4%)	11 (52,4%)
Triarterial	9 (40,9%)	5 (23,8%)
TCE	0 (0%)	2 (9,5%)
Escore *Syntax* médio	27,2	10,9

*ICP: intervenção coronária percutânea; TC: tronco da artéria coronária esquerda.*

As justificativas para encaminhamento dos pacientes para TCO ou ICP são trazidas nas Tabelas 2 e 3 , respectivamente.

**Tabela 2:** – Justificativa para manutenção em tratamento clínico otimizado

Justificativa	n (%)
Revisão de gravidade anatômica	7 (31,8%)
Baixa carga isquêmica	6 (27,2%)
Anatomia desfavorável	6 (27,2%)
Elevado risco cirúrgico	3 (13,6%)

**Tabela 3:** – Justificativa para opção por tratamento percutâneo

Justificativa	n (%)
Passível de ICP	15 (71,4%)
Revisão de gravidade anatômica	5 (23,8%)
Elevado risco cirúrgico	1 (4,8%)

*ICP: intervenção coronária percutânea.*

### Seguimento de um ano

Os pacientes tiveram seguimento médio de 13 meses. Dois episódios de IAM ocorreram entre pacientes encaminhados para realização de ICP, e resultaram em óbito: uma mulher apresentou morte súbita precedida de precordialgia típica antes da realização da ICP e um homem apresentou IAM periprocedimento evoluindo com choque cardiogênico refratário. Os demais pacientes não apresentaram eventos cardiovasculares maiores.

## Discussão

Devido à importante redução do número de cirurgias durante a pandemia de COVID-19, causada pela falta de leitos de UTI e afastamento dos servidores hospitalares^1 , 2^ foi necessária a revisão da lista de CRM. O risco de infecção intra-hospitalar também foi relevante, por aumentar significativamente a morbimortalidade.^3 , 4^ Baseado em diretrizes internacionais, o HT modificou a estratégia de tratamento de 12% dos pacientes, que foram acompanhados por um ano.

Alguns dos pacientes incluídos no nosso estudo apresentavam indicação mais sedimentada de revascularização cirúrgica.^5 - 8^ Contudo, considerando o risco de um tempo indeterminado, a espera do tratamento cirúrgico e a expertise da equipe de cardiologia intervencionista do nosso serviço, o HT optou pela mudança no tratamento.

Estudos recentes corroboram essa ideia. Uma coorte de 215 pacientes de 45 centros do Reino Unido, inicialmente incluídos em fila de CRM, foi submetida à ICP em função do longo tempo de espera. No seguimento de 30 dias, apresentaram desfechos clínicos semelhantes aos tradicionalmente encontrados em pacientes submetidos à CRM.^9^O fato dos desfechos do TCO serem comparáveis aos do tratamento invasivo em pacientes estáveis com carga isquêmica relevante^10^ foi importante para mudança da estratégia nesse grupo.

A relevância do trabalho em equipe no manejo da DAC complexa tem sido demonstrada.^11 , 12^As recomendações de tratamento da DAC multiarterial do cardiologista intervencionista isoladamente e do HT são discordantes em um número significativo de casos.^13^ Além disso, seguimentos de longo prazo de pacientes cuja estratégia de tratamento foi definida por HT tem demonstrado tomadas de decisão apropriadas e personalizadas, com desfechos favoráveis.^14^

Nosso estudo tem diversas limitações. O número limitado de pacientes incluídos em nosso estudo pode restringir a generalização dos dados. Entretanto, listas cirúrgicas devem ser idealmente pequenas, para não permitir tempo prolongado de espera. Um maior tempo de seguimento desses pacientes poderia trazer resultados mais fidedignos a respeito dos desfechos encontrados. Estudos multicêntricos com amostras maiores e desenhos adequados são necessários para elucidar o real impacto da exclusão de pacientes da fila de CRM por HT com base nas atuais evidências disponíveis.

No presente estudo, a reavaliação de pacientes em fila de CRM por HT durante a pandemia da COVID-19 permitiu a redução de 12% do número de indicações de tratamento cirúrgico, sendo possível a mudança para TCO ou ICP, de acordo com recentes diretrizes e estudos. Entre os pacientes que tiveram sua estratégia terapêutica modificada, observamos uma excelente sobrevida livre de eventos em um ano.
