# Supplementary material for: Mudança de Conduta em Pacientes de Fila de Cirurgia de Revascularização Miocárdica durante Pandemia de COVID-19: Seguimento de um Ano
Source: Arq Bras Cardiol. 2023 Apr 12;120(4):e20220582. [Article in Portuguese] doi: 10.36660/abc.20220582 (PMC10263414; doi:10.36660/abc.20220582)
Supplement: Translation

# Translation: Change of Strategy in Coronary Artery Bypass Graft Surgery Waiting List during the COVID-19 Pandemic: One-Year Follow-Up

**Authors:** Franc Jorge Sampaio Santos Pereira, Marília Prudente Menezes, Gabriela Carolina Santamaria Naranjo, José Henrique Herrmann Delamain, José Ribamar Costa, Mario Issa, Vivian Lerner Amato, Fausto Feres, Pedro Silvio Farsky

**Affiliations:** 1 Brazil Instituto Dante Pazzanese de Cardiologia, São Paulo, SP – Brazil

**Keywords:** Coronary Artery Disease/surgery, Myocardial Revascularization, Intervention Coronary Percutaneous, Myocardial Infarction/complications, COVID-19, Pandemic, Waiting Lists, Technology Applied to Waiting Lists

## Introduction

The COVID-19 pandemic led to a significant occupation of intensive care beds, causing the suspension of elective cases of coronary artery bypass grafting (CABG).

At the same time, the evolution of techniques for the percutaneous treatment of coronary artery disease (CAD) has expanded the possibilities for this modality, and published studies have not shown a reduction in outcomes in stable patients with moderate/high ischemic burden under optimized clinical treatment (OCT).

In this context, all patients included in the CABG queue of a tertiary cardiology hospital had their indications reviewed by a Heart Team (HT) in order to assess a possible change in strategy for percutaneous coronary intervention (PCI) or maintenance on OCT. We present the clinical and angiographic characteristics, the reasons for changing the therapeutic strategy, as well as the clinical evolution after one year of follow-up.

## Methods

All patients included in a CABG queue between June 2020 and April 2021 were reviewed by a HT with senior clinical, hemodynamics and surgery specialists in order to assess change in treatment strategy. Patients with an angiographic study performed more than one year prior had their study repeated, as well as those with poor image quality, or significant change in symptomatology. Patients with few symptoms or with doubtful indication of coronary artery bypass grafting were tested to quantify the ischemic area.

Patients whose surgical indication was changed to another treatment modality were included in this study. They were examined for comorbidities, coronary anatomy and surgical risk, as well as the reasons for the change in management and clinical evolution in the first year. Patients were followed up by a team dedicated to the study and maintained on optimal medical treatment.

Regarding clinical outcomes, the incidence of all-cause mortality, cardiovascular mortality, acute myocardial infarction (AMI), hospitalization for cardiovascular causes, relevant bleeding, and stroke was evaluated.

## Results

Of the 357 patients included in the CRM queue between June 2020 and April 2021, 43 (12.0%) were excluded by the HT, with 21 undergoing PCI and 22 maintained on OCT. Patients referred for PCI had a higher prevalence of diabetes (57.1% vs. 36.3%) and previous AMI (80.9% vs. 59%). Those maintained on OCT had a higher prevalence of previous stroke (13.6% vs. 0%) and higher surgical risk according to the STS score (1.12% vs. 0.68%). Five patients (11.6%) had limiting symptoms: three in NYHA III were maintained on OCT, one because of the high surgical risk and the other two because chronic obstructive pulmonary disease was identified as the cause of dyspnea; two patients with CCS 3 angina were referred for PCI.

Angiographic characteristics are presented in Table 1 .

**Table 1:** – Angiographic characteristics of patients maintained on optimized clinical treatment and undergoing PCI

Affected artery(ies)	Clinical (n = 22)	PCI (n = 21)
Anterior descending artery	3 (13.6%)	3 (14.2%)
Double vessel	10 (45.4%)	11 (52.4%)
Triple vessel	9 (40.9%)	5 (23.8%)
LMCA	0 (0%)	2 (9.5%)
Average Syntax score	27.2	10.9

*PCI: percutaneous coronary intervention; LMCA: left main coronary artery.*

The justifications for referring patients to OCT or PCI are shown in Tables 2 and 3 , respectively.

**Table 2:** – Justification for maintaining on optimized clinical treatment

Justification	n (%)
Anatomical severity review	7 (31.8%)
Low ischemic burden	6 (27.2%)
Unfavorable anatomy	6 (27.2%)
High surgical risk	3 (13.6%)

**Table 3:** – Justification for choosing percutaneous treatment

Justification	n (%)
Eligible for PCI	15 (71.4%)
Anatomical severity review	5 (23.8%)
High surgical risk	1 (4.8%)

*PCI: percutaneous coronary intervention.*

### One-year follow-up

Patients had a mean follow-up of 13 months. Two AMI episodes occurred among patients referred for PCI and resulted in death￼: one woman had sudden death preceded by typical chest pain before PCI and one man had periprocedural AMI progressing to refractory cardiogenic shock. The remaining patients did not have major cardiovascular events.

## Discussion

Due to the significant reduction in the number of surgeries during the COVID-19 pandemic, caused by the lack of ICU beds and the number of hospital personnel on leave,^1 , 2^revision of the CABG waiting list was necessary. The risk of in-hospital infection was also relevant, as it significantly increased morbidity and mortality.^3 , 4^Based on international guidelines, the HT changed the treatment strategy of 12% of patients, who were followed up for one year.

Some of the patients included in our study had a more solid indication for coronary artery bypass grafting.^5 - 8^However, considering the risk of an indefinite period of time, the wait for surgical treatment and the expertise of the interventional cardiology team at our service, the HT opted for a change in treatment.

Recent studies corroborate this idea. A cohort of 215 patients from 45 centers in the United Kingdom, initially included in the CABG waiting list, underwent PCI due to the long waiting time. In the 30-day follow-up, they presented clinical outcomes similar to those traditionally found in patients undergoing CABG.^9^

The fact that OCT outcomes were comparable to those of invasive treatment in stable patients with relevant ischemic burden^10^was important for changing the strategy in this group.

The relevance of teamwork in the management of complex CAD has been demonstrated.^11 , 12^Treatment recommendations for multivessel CAD by the interventional cardiologist alone and by the HT are in disagreement in a significant number of cases.^13^Furthermore, long-term follow-up of patients whose treatment strategy was defined by the HT has demonstrated appropriate and personalized decision-making, with favorable outcomes.^14^

Our study has several limitations. The limited number of patients included in our study may restrict data generalizability. However, surgery waiting lists should ideally be short to avoid long waiting times. A longer follow-up time for these patients could bring more reliable results regarding the outcomes found. Multicentric studies with larger samples and adequate designs are needed to elucidate the real impact of HT exclusion of patients from the CABG waiting list based on the current available evidence.

In this study, the reassessment of patients queuing for CABG due to HT during the COVID-19 pandemic allowed a 12% reduction in the number of surgery indications, making it possible to switch to OCT or PCI, according to the latest guidelines and studies. Among patients who had their therapeutic strategy modified, we observed excellent event-free survival at one year.
